# Optimization and Characterization of Phenolic Extraction Conditions and Antioxidant Activity Evaluation of *Adenanthera pavonina* L. Bark

**DOI:** 10.3390/plants12223902

**Published:** 2023-11-19

**Authors:** Syeda Nurunnesa Begum, Mobarok Hossain, Md. Adnan, Chowdhury Habibur Rahaman, Arif Reza

**Affiliations:** 1 Ethnopharmacology Laboratory, Department of Botany, Visva-Bharati University, Santiniketan 731235, West Bengal, India; 03333321808@visva-bharati.ac.in; 2 Department of Applied Geosciences, GZG—University of Göttingen, Goldschmidtstraße 3, 37077 Göttingen, Lower Saxony, Germany; mobarok.hossain@uni-goettingen.de; 3 Department of Molecular Pharmaceutics, College of Pharmacy, University of Utah, Salt Lake City, UT 84112, USA; md.adnan@utah.edu; 4 New York State Center for Clean Water Technology, Stony Brook University, Stony Brook, NY 11794, USA; 5 School of Marine and Atmospheric Sciences, Stony Brook University, Stony Brook, NY 11794, USA; 6 Department of Environmental Science, College of Agricultural Sciences, IUBAT—International University of Business Agriculture and Technology, Dhaka 1230, Bangladesh

**Keywords:** *Adenanthera pavonina* bark, optimization, phenolic profiling, high-performance liquid chromatography, antioxidant activity

## Abstract

The presence of high levels of secondary metabolites in medicinal plants can significantly influence the progress of drug development. Here, we aimed to maximize phenolic extraction from *Adenanthera pavonina* L. stem bark using various solvents such as ethyl acetate, methanol, petroleum ether, and chloroform. A response surface method (RSM) with a central composite design (CCD) statistical technique was applied to optimize the extraction process, employing three important extracting parameters such as extraction time (h), temperature (°C), and solvent composition (% *v/v* of methanol/water) to obtain the highest phenolic content. Total phenolic content (TPC) and antioxidant activity (IC_50_ of extract’s DPPH radical scavenging activity) were used as response variables to find the influence of these extracting parameters. Among the various solvents used, methanol extract showed the highest contents of phenolics and the maximum level of antioxidant activity with a lower IC_50_ value. The notable TPC and IC_50_ value of the extract’s DPPH radical scavenging capacity were found to be 181.69 ± 0.20 mg GAE/g dry tissue and 60.13 ± 0.11 mg/mL, respectively, under the optimal conditions with a solvent composition of 71.61% (*v/v*) of methanol/water, extraction temperature of 42.52 °C, and extraction time of 24 h. The optimized extract of *A. pavonina* stem bark was further subjected to HPLC analysis, where six phenolic compounds, including coumarin, p-coumaric acid, chlorogenic acid, sinapic acid, gallic acid, and caffeic acid, were identified along with their respective quantities. Overall, the findings of this study uncover a low-cost analytical model for maximizing phenolic extraction from *A. pavonina* bark with enhanced antioxidant activity.

## 1. Introduction

*Adenanthera pavonina* L. is a perennial and tall tree in the Leguminosae family that is found in tropical and subtropical areas around the world. This tree is also known as the red bead tree worldwide. Traditionally, different parts of this plant have been used to treat an extensive range of conditions including hypertension, boils, diabetes, diarrhea, arthritis, cholera, asthma, stomach bleeding, fever, indigestion, epilepsy, vomiting, pulmonary infections, and cancer [1,2,3]. Besides these, bark and leaves are also used as a remedy for ulcers, gout, dysentery, hematemesis, and rheumatism [4]. Several studies have investigated the phytochemical and pharmacological possibilities of various parts of *A. pavonina* in the past [5,6,7,8]. Pharmacological investigations have shown that different parts of this plant have antinociceptive, anti-inflammatory, analgesic, anti-diarrheal, antimicrobial, antioxidant, anti-hyperglycemic, and hypolipidemic properties [2,7,9]. Phytochemical studies have demonstrated that different parts of this plant contain a wide array of chemical compounds including phenolics, flavonoids, alkaloids, steroids, saponins, triterpenoids, and glycosides [7,10].

The phenolic compounds found naturally in *A. pavonina* L. were suggested to be the major contributors to the antioxidant activities of the plant [11,12]. In general, the existence of hydroxyl groups is mainly responsible for the antioxidant capacity of phenolic compounds, which possess the ability to donate electrons [13]. These phenolic compounds also boost the antioxidant activities of cells by stimulating the synthesis of endogenous antioxidant molecules like glutathione peroxidase, superoxide dismutase, and catalase within the cell and inducing their defense mechanisms through the regulation of multiple signaling pathways [14,15,16]. 

Several existing studies have revealed that phenolic substances can suppress the generation of free radicals, break down peroxides and inactive metal ions, and hinder the process of oxygen scavenging within biological systems that ultimately defend against many oxidative diseases [17]. Thus, phenolic compounds from medicinal plants have recently gained extensive attention because of their plentiful pharmacological actions such as preventing neurodegenerative diseases, body inflammation, cancers, diabetes, osteoporosis, and cardiovascular problems [14]. Given the intrinsic utility and broad spectrum of potential uses in the pharmaceutical sector, it is crucial to concentrate on phenolic extraction. This is one of the most important steps because it is the initial step in the process of isolating bioactive compounds from plant parts.

Due to the diverse sources of phenolics from a particular plant, and the complex structure of these compounds, the extraction methods for phenolic compounds vary. Therefore, an ideal extraction method for a particular source, rich or poor in phenolic substances, has to be individually designed and optimized so that the maximum amount of phenolic compounds can be gained with minimal changes to the functional properties [18]. The extraction of phenolic compounds from a plant is generally influenced by several factors such as solvent–solid ratio, extraction time, solvent composition, particle size, solvent polarity, extraction temperature, and pH [19]. Conventional phenol extraction methods utilize a single factor or variable at a time in an experimental setup and this is called the one-variable-at-a-time technique. The major limitation of this method is that there is no scope to study the interactive effects of all the variables considered in a single experimental setup. Several other drawbacks attached to this one-variable technique are poor extraction efficiency, lengthy extraction time, and loss of thermolabile compounds [20]. To address these issues, the current work utilized the response surface methodology (RSM), a multivariate statistical technique, described by Box and Wilson, 1951 [21], to optimize the extraction procedures. The response surface methodology (RSM) is an effective statistical and mathematical technique that widely relates multiple parameters or variables to attain the optimal system efficiency for maximal bioactive phenolic extraction [22]. 

The current study highlights how to maximize the efficiency of phenolic extraction considering some important factors (solvent composition (*v/v*), extraction time (h), and extraction temperature (°C)) to gain the maximum possible phenolic content and antioxidant activity from the bark part of *A. pavonina,* since its stem bark contains a high amount of phenolics. The literature review revealed that no prior data focused on the optimization of the extraction of maximum phenolic compounds from the stem bark of *A. pavonina*. Therefore, the design of an optimized extraction method was performed for the first time to obtain a higher yield of phenolic compounds from *A. pavonina* bark. The RSM-based optimized extraction procedure was used to enhance the extraction efficiency, lower the cost, and reduce the time for various experiments. The present study stresses the effectiveness of the RSM technique in optimizing the extraction parameters for the highest phenolic content and provides an assessment of the antioxidant activity of the bark part of *A. pavonina* including phenolic compound characterization using the HPLC (high-performance liquid chromatography) method.

## 2. Results and Discussion

### 2.1. Impact of Various Extraction Solvents on TPC and Antioxidant Activity

Phenolic compounds are one type of secondary metabolites that are synthesized by plants. These compounds are attributed to the antioxidant properties of plants that neutralize free radicals through multifunctional properties such as singlet oxygen quenching, hydrogen donating, reducing foreign elements, and metal chelating [23]. Nowadays, scientists are paying attention to isolating phenolic compounds from plants so that they can be used as a natural antioxidant supplement in several dietary products. Importantly, different components of a plant possess a reservoir of bioactive substances that encompass possible chemical groups. These compounds frequently exhibit protective properties against cellular oxidative damage in plants and animals.

A varied range of phenolic content is found in different plant taxa. It is essential to employ effective extraction methods that give higher yields with minimal changes to the functional properties of phenolic compounds present in the extract. These methods include maceration, percolations, infusion, decoctions, supercritical fluid extraction, microwave-assisted solvent extraction, and ultrasonic-assisted extraction, all of which require appropriate solvents for optimal results. Significantly, plant-based compounds have a wide range of chemical properties and polarities that pose challenges in terms of solubility in specific solvents. Hence, it is imperative to investigate a range of solvents with varying polarity in order to extract potential bioactive components from the plant [24]. In our study, the bark of *A. pavonina* was macerated using ethyl acetate, methanol, petroleum ether, and chloroform to obtain the highest total phenolic content and antioxidant activity.

**Figure 1:**
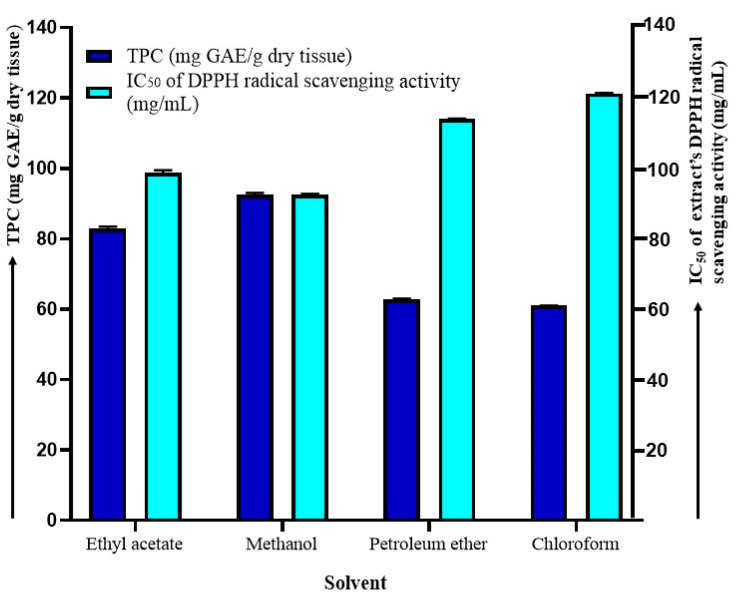
Effect of different solvents extracts on the extraction of TPC (total phenolic content, mg GAE/g dry tissue) and antioxidant activities (IC_50_ of extract’s DPPH radical scavenging activity, mg/mL) from the *A. pavonia* stem bark.

Figure 1 reveals that different solvent extracts had different total phenolic contents and variable antioxidant activities. The maximum TPC and enhanced antioxidant activity (in terms of IC_50_ values as depicted from the DPPH activity) estimated in the methanol extract were 92.51 ± 0.51 mg GAE/g dry tissue and 92.45 ± 0.334 mg/mL, respectively. IC_50_ standard (ascorbic acid) was found to be 59.82 ± 0.6 mg/mL. In this investigation, the order of the yield of TPC and antioxidant activity by using different solvents was found to be in the following descending order: methanol > ethyl acetate> petroleum ether > chloroform (Figure 1). 

The chemical components of a plant may display either polar or nonpolar characteristics. Specifically, phenolic compounds are known to include many hydroxyl groups, which facilitate their solubility in polar solvents. Hydroxyl groups in phenolic compounds can develop hydrogen bonds with the electronegative oxygen of polar solvents such as methanol and ethanol. Researchers have determined that strongly polar solvents like methanol are more effective in extracting phenolics from plant-based sources [25]. The methanol extract may have exhibited the highest TPC due to its elevated polarity index. Moreover, the significant TPC observed in the methanol extract could perhaps be attributed to the existence of methyl radicals, which have the ability to readily combine with phenolic compounds, facilitating effective solvation [26]. Furthermore, the methanol extract exhibited superior extraction of TPC from *A. pavonina* bark, validating the efficacy of this solvent for extracting phenolic compounds. Our findings support the claim that the methanol solvent shows potential for the extraction of maximum phenolic compounds with nominal changes to their functional properties. 

Plant extracts rich in phenolics and other antioxidant phytochemicals exhibited a significantly higher value of free radical scavenging [27]. DPPH, also known as 2,2-diphenyl-1-picrylhydrazyl, is an organic nitrogen radical, which has an unpaired valence electron at one atom of its nitrogen bridge that serves as a free radical. It has been widely used for testing the preliminary antioxidant activity of plant extracts. Here, methanol and ethyl acetate extracts showed promising DPPH radical scavenging activity with a lower IC_50_ similar to the TPC result. It was noteworthy that there was a positive correlation between antioxidant activity and the level of total phenolic content. Different phenolic groups have different hydroxyl groups, which are responsible for their radical scavenging activity. Two main mechanisms by which antioxidants manifest these properties are free radical inactivation and electron transfer [28]. Therefore, it is possible that the increased antioxidant activity found in our study can be linked to the presence of phenolics in higher amounts in the stem bark extracts.

### 2.2. Optimization through Response Surface Methodology (RSM) Model

Table 1 displays the results of TPC extraction and the IC_50_ of the extract’s DPPH radical scavenging activity obtained during extraction using CCD. Based on the experimental outcomes, the coded versions of the subsequent second-order polynomial quadratic equations (Equations (1) and (2)) were developed:

TPC (mg GAE/g dry tissue) = 180.83 + 0.92*x*_1_ − 14.88*x*_2_
*+* 13.28*x*_3_ − 20.29*x*_1_^2^ − 24.67*x*_2_^2^ − 14.69*x*_3_^2^ − 0.01*x*_1_*x*_2_ + 0.22*x*_1_*x*_3_ − 4.99*x*_2_*x*_3_ (1)

IC_50_ of DPPH radical scavenging activity (mg/mL) = 65.00 − 1.30*x*_1_
*+* 1.83*x*_2_ − 4.84*x*_3_ + 13.35*x*_1_^2^ + 12.25*x*_2_^2^ − 4.52*x*_3_^2^ − 0.91*x*_1_*x*_2_ − 0.81*x*_1_*x*_3_ + 1.12*x*_2_*x*_3_ (2)

**Figure 2:**
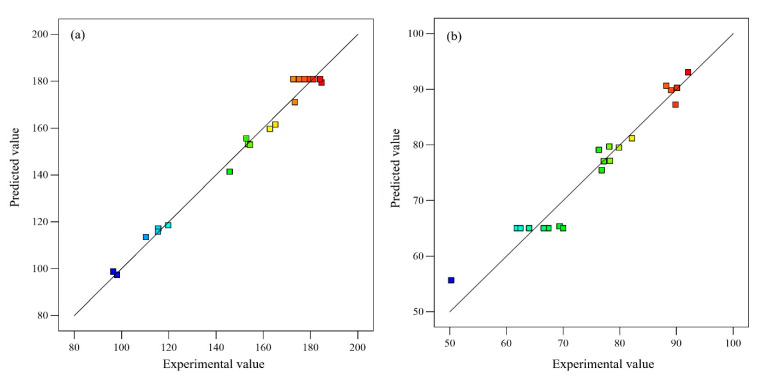
Linear fit for predicted values against experimental observations: (**a**) TPC and (**b**) IC_50_ of extract’s DPPH radical scavenging activity.

Multiple regression analysis was employed to compute the model coefficients and the fitness of the developed models was assessed by examining the correlation coefficient of the model (R^2^) and the adjusted R^2^ (R^2^_adj_) and predicted R^2^ (R^2^_pre_) values. The R^2^_adj_ accounts for changes in the R^2^ value, which enhances the model’s ability to explain the numerical aspects of the data points. On the other hand, R^2^_pre_ gauges the ability of a regression model to predict responses using additional observations [29]. Here, the Design Expert (version 13.0.5, Stat-Ease Inc., Minneapolis, MN, USA) software was used to derive both R^2^_adj_ (0.97 and 0.90) and R^2^_pre_ (0.93 and 0.72) values for TPC extraction and the IC_50_ of the extract’s DPPH radical scavenging activity, respectively, and exhibited strong concurrence. Furthermore, the R^2^ values of 0.98 and 0.94 signify that the model-predicted values are in good agreement with experimental observations. ANOVA was used to assess the statistical significance of the developed second-order polynomial quadratic equations (Equations (1) and (2)). The results of ANOVA are presented in Table 1 and Table 2. Key model indicators such as Fisher’s F-value, lack of fit, and acceptable accuracy are crucial in establishing the significance, capability, and reliability of RSM-based models [30]. As shown in Table 1 and Table 2, the computed Fisher’s F-values (85.75 and 20.66), with a significance level of *p* < 0.0001, effectively affirmed the suitability of the suggested regression models in projecting the results [31]. The lack-of-fit value aids in figuring out the relative distinction between residual errors and pure errors in an executed experimental design, and its insignificance is imperative to validate the credibility of the constructed model [32]. In the present study, the lack-of-fit value was found to be insignificant in both quadratic equations (*p* > 0.1), denoting the viability of the developed models. Moreover, the values of adequate precision (24.92 and 14.92) were higher than 4, implying the capability of the constructed models to effectively govern the design space [33]. In addition, the capability of the suggested second-order polynomial quadratic models to fit the TPC extraction and the IC_50_ of the extract’s DPPH radical scavenging activity was confirmed by plotting the projected values against the actual experimental data points (Figure 2). The figure displays a linear distribution of predicted and observed values in both cases, indicating the goodness of fit and assessment potential of the proposed models during the experiment.

**Figure 3:**
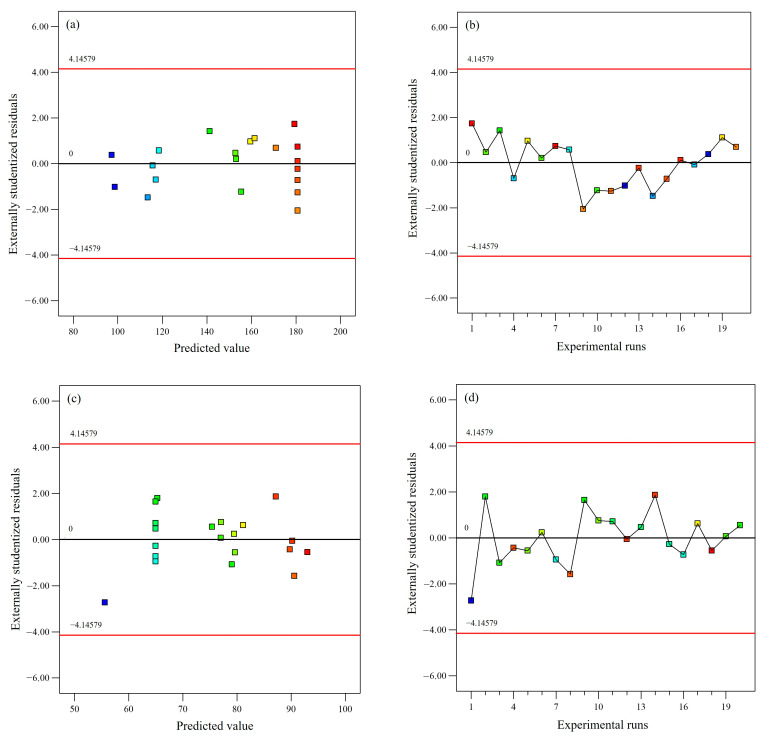
Diagnostic plots for TPC (externally studentized residual versus (**a**) predicted value and (**b**) experimental runs) and IC_50_ of extract’s DPPH radical scavenging activity (externally studentized residual versus (**c**) predicted value and (**d**) experimental runs).

**Figure 4:**
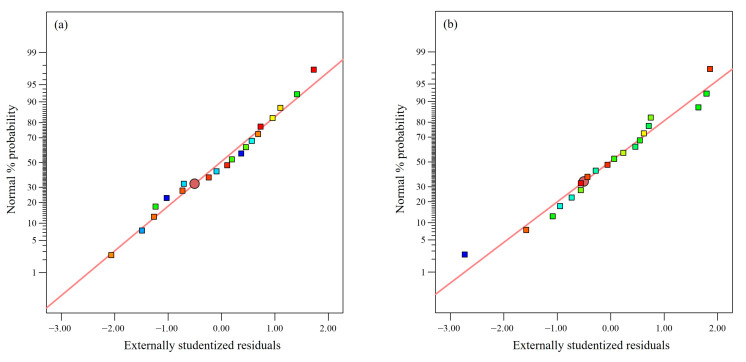
Normal probability plot of externally studentized residuals for (**a**) TPC and (**b**) IC_50_ of extract’s DPPH radical scavenging activity.

Moreover, the diagnostics plots such as externally studentized residuals versus predicted values and externally studentized residuals versus experimental runs did not show any outliers (Figure 3), hence epitomizing the developed models’ reliability with the obtained experimental values for both TPC extraction and IC_50_ of the extract’s DPPH scavenging activity. The normal plot of residuals elucidated the normal residual distribution following a straight-line pattern and further confirmed the rational agreement between the predicted and actual values (Figure 4). Overall, the statistical interpretation of the results indicates the suitability of the established polynomial quadratic models in forecasting the TPC content and IC_50_ of the extract’s DPPH scavenging activity values.

#### 2.2.1. Effects of Operational Parameters on TPC Extraction and IC_50_ of Extract’s DPPH Radical Scavenging Activity

**Figure 5:**
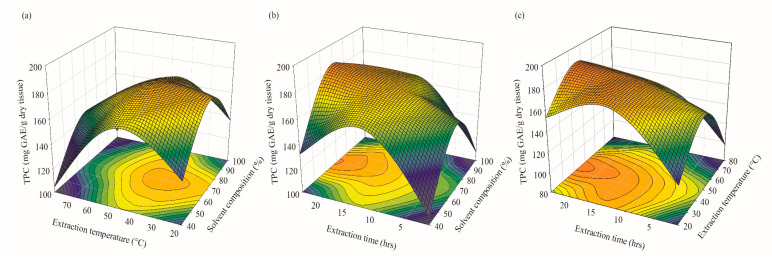
Three-dimensional surface and contour plots for TPC extraction from *A. pavonina* bark: (**a**) extraction temperature (°C) × solvent composition (%), (**b**) extraction time (h) × solvent composition (%), and (**c**) extraction time (h) × extraction temperature (°C).

**Figure 6:**
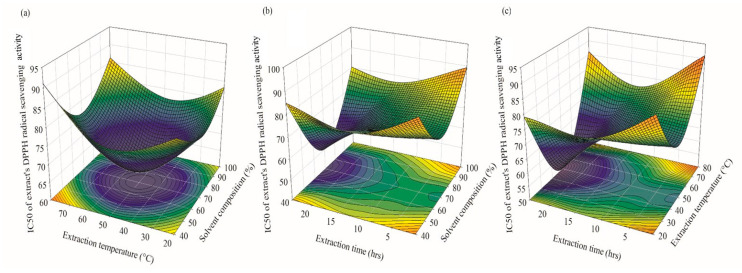
Three-dimensional surface and contour plots for IC_50_ of extract’s DPPH radical scavenging activity: (**a**) extraction temperature (°C) × solvent composition (%), (**b**) extraction time (h) × solvent composition (%), and (**c**) extraction time (h) × extraction temperature (°C).

The linear, quadratic, and interaction impacts of the specified independent operational factors on TPC extraction from *A. pavonina* bark and the IC_50_ of the extract’s DPPH radical scavenging activity are explained by the regression analysis in Table 1 and Table 2, respectively. The principal and cross-interactive effects of independent factors on the target responses are also represented via the 3D response graph and contour plots (Figure 5 and Figure 6).

Table 2 shows that the TPC extraction from *A. pavonina* bark is controlled by the extraction temperature and time at both linear and quadratic levels (*p* < 0.001), while the extraction solution significantly affected the TPC extraction process only at the quadratic level (*p* < 0.0001). In terms of the interactive impacts of all investigated independent factors, extraction temperature and time showed substantial positive effects on TPC extraction (*p* < 0.05). The aforementioned results are validated further by the 3D responsive surface and contour diagrams shown in Figure 5 and Figure 6 and supported by many previous studies [34,35,36]. It was evidenced that the majority of the phenolic compounds are heat-sensitive in nature and can be oxidized or degraded easily [37,38]. Therefore, a high temperature together with a prolonged extraction time can lead to the decomposition of phenolic compounds [34]. Furthermore, Jahromi [35] and Shi et al. [36] also highlighted the significance of extraction temperature and time for TPC extraction from plant materials.

Extraction time showed a significant impact at a linear level on the IC_50_ of the extract’s DPPH scavenging activity (*p* < 0.05). Earlier studies also signified the effect of extraction time on the increased antioxidant activity of extracts [39,40].

On the other hand, solvent composition and extraction temperature showed profound influences at quadratic levels (*p* < 0.001) (Table 3). In the case of interactive effects, none of the process parameters influenced the IC_50_ of the extract’s DPPH radical scavenging activity (*p* > 0.05). To sum up, the findings described above provide useful insights regarding the impact of independent operational factors on the TPC extraction efficiency from *A. pavonina* bark and the IC_50_ in the extract’s DPPH radical scavenging test.

#### 2.2.2. Optimization and Validation of the Extraction Process

The independent parameters that have insightful influences on the performance of the process are generally selected from the prevailing options. The extraction of phenolic compounds and the antioxidant potential of the extracts are generally influenced by extraction parameters such as solvent composition, temperature, and time. The antioxidant potential of plants is strongly correlated with phenolic compounds because of their hydroxyl groups at ortho and para positions contributing to the antioxidant property. In the present work, the extraction of phenolics was influenced by an optimized binary solvent composition, temperature, and time. Methanol was the solvent of choice for extracting phenolics due to its high polarity. Its superiority was also well documented in previous studies [25]. 

An optimized alcohol (methanol) and water ratio is a key factor in the extraction of phenolic compounds. Water helps to swell plant tissues, while alcohol helps to dissolve and recover phenolic compounds. The TPC content and DPPH scavenging activity (lower IC_50_ value) initially increased with increasing methanol concentration until reaching a maximum level and then started to decrease after a certain concentration. High concentrations of methanol can cause molecule congestion, which hinders the mass transfer and ultimately reduces the extraction of phenolic compounds [41]. Furthermore, an increase in extraction temperature enhances the exaction of phenolic compounds. High temperatures during extraction promote polysaccharides on the cell wall, which aid in dispersing the solvent used for extraction by undermining the strength of the cell wall. However, if the extraction process is performed at temperatures beyond the optimum, the phenolic yield may be decreased because it causes a loss of solvent and accelerates phenolic oxidation or degradation [42,43]. Moreover, extraction time governs the interaction between the extraction medium (aqueous methanol) and plant material, and a longer extraction time facilitates better extraction [34]. Therefore, it is of utmost importance to ascertain the best possible conditions to confirm effective and efficient process performance during extraction.

**Figure 7:**
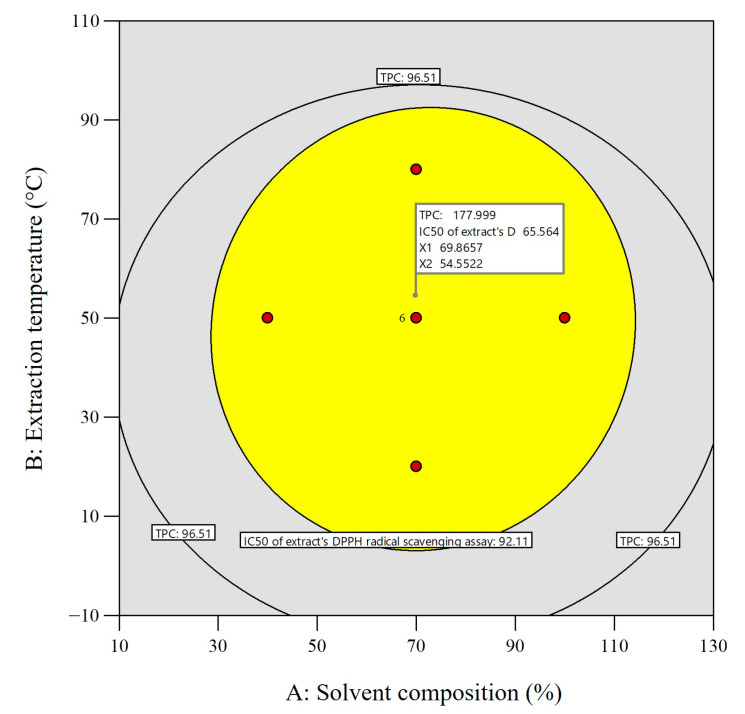
Overlay plot showing the ideal zone with an extraction time of 12.5 h.

The targeted levels for the independent operating variables and target responses were set to ‘within the range’ and ‘maximum’, respectively, in this investigation. All independent factors and target responses were given equal weightage. Table 4 shows the most appropriate operating conditions for TPC extraction from *A. pavonina* bark, as well as for the IC_50_ of the extract’s DPPH scavenging activity. The optimized conditions were then independently tested in triplicate, and their validity was confirmed by comparing the results to those predicted by the model. High TPC and IC_50_ of the extract’s DPPH radical scavenging activity of 181.69 ± 0.20 mg GAE/g dry tissue and 60.13 ± 0.11 mg/mL, respectively, were obtained under the optimized conditions (solvent composition 71.61%, extraction temperature 42.52 °C, and extraction time: 24 h), which were found to be fairly close to the RSM-model-predicted values and within the 95% confidence level (Table 4). Moreover, the optimal operating conditions were justified by means of the overlay plots. The yellow zone in the overlay plot indicated the optimal region as a design space. The selected values for the TPC and IC_50_ of the extract’s DPPH scavenging activity were 177.99 mg GAE/g dry tissue and 65.56 mg/mL, at the solvent composition, extraction temperature, and extraction time of 69.87%, 55.55 °C, and 12.5 h, respectively, and are symbolized by a flag (Figure 7). Through the optimization method, an increased extraction of TPC and IC_50_ of the extract’s DPPH radical scavenging activity from *A. pavonina* bark was achieved by using methanol as a solvent compared to a non-optimized condition (92.51 ± 0.51 mg GAE/g dry tissue and 92.45 ± 0.334 mg/mL). A previous study revealed that the TPC extracted from the bark of *A. pavonina* using a methanol solvent in non-optimized conditions is 8.51 mg/g [3]. Rodrigo et al. [44] assessed the antioxidant activity (DPPH activity) of non-optimized methanolic extracts of *A. pavonina* bark, and it was 70.3 ± 0.5 mg/mL. These findings imply that the optimization of operational conditions ensures maximum TPC extraction from *A. pavonina* bark with higher antioxidant activity as compared to a non-optimized extract.

### 2.3. Identification and Quantification of Phenolic Compounds

**Figure 8:**
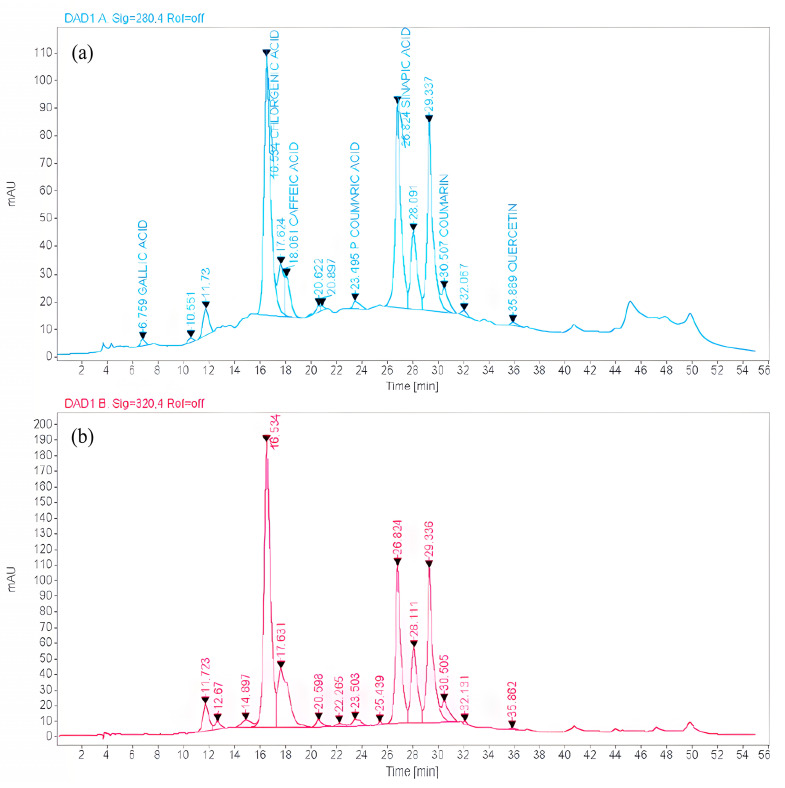
HPLC chromatogram of (**a**) standard phenolic compounds and (**b**) optimized extract of *A. pavonina* stem bark.

To characterize the phytochemicals from the optimized phenolic extract, ten standards of phenolic compounds (kaempferol, gallic acid, syringic acid, chlorogenic acid, catechin hydrate, caffeic acid, p-coumaric acid, sinapic acid, coumarin, and quercetin) were used for HPLC analysis. The HPLC examination of the *A. Pavonina* optimized stem bark extract indicated the presence of a total of six phenolic components, which have been identified and quantified, namely chlorogenic acid (117.526 mg/g dry tissue), gallic acid (0.042 mg/g dry tissue), caffeic acid (5.809 mg/g dry tissue), sinapic acid (64.779 mg/g dry tissue), p-coumaric acid (0.487 mg/g dry tissue), and coumarin (1.523 mg/g dry tissue). The identified six phenolic compounds have predominantly hydrophilic characteristics. The present study utilized the RSM model to determine the optimal solvent for extracting greater amounts of phenolic compounds, with methanol being identified as the most effective solvent in combination with water. Among the identified six compounds from the bark extract, chlorogenic acid, and sinapic acid are the prominent phenolic acids, which have well-documented antioxidant, anti-inflammatory, anticancer, and antibacterial effects [45,46] (Table 5). Other phenolic compounds such as gallic acid, caffeic acid, p-coumaric acid, and coumarin were found in subordinate quantities. All these acknowledged compounds have well-documented antioxidant, anti-inflammatory, anticancer, and antibacterial effects [45,46,47,48,49,50,51,52] (Table 5). Thus, the stem bark of *A. pavonina* can also be recognized as a new important source of phenolic compounds. Previously, scientists [10,11] demonstrated that *A. pavonina* has some antioxidant activities and the present study evidenced that those activities are mainly due to the presence of phenolic compounds like chlorogenic acid and sinapic acid since they were found in the highest concentration in the optimized bark extract (Figure 8). 

## 3. Materials

### 3.1. Plant Collection and Sample Preparation

**Figure 9:**
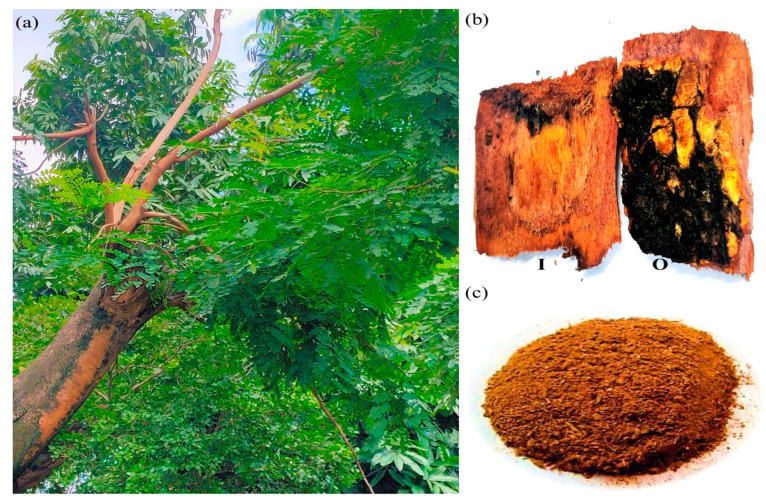
(**a**) The tree of *Adenanthera pavonina* L., (**b**) stem bark (inner (I) and outer (O) surfaces), (**c**) powder of stem bark.

Identification of the plant (Figure 9a) was performed with the help of different floras [53] and authenticated by consulting with plant taxonomists. A standard website, Plants of the World Online [54] was also followed for the nomenclature verification of the identified plant species. The fresh stem bark parts of *Adenanthera pavonina* L. were collected in December 2022 from Santiniketan, Birbhum (23°40′52″ N, 87°40′20″ E), a popular semi-arid tropical region of West Bengal, India (Figure 9b). The harvested plant materials were adequately rinsed with tap water to eliminate any remaining dust before being chopped into small fragments, shade-dried, and finely powdered using an electric grinder. The crude powder was then stored in a tightly sealed container at 4 °C until it was used for further analyses (Figure 9c). According to accepted herbarium procedures [55], collected plant specimens were also stored as specimen vouchers in the Department of Botany, Visva-Bharati, Santiniketan, India, for future use (Specimen number: SN Begum 6, India, West Bengal, Birbhum: Santiniketan, 28 December 2022).

### 3.2. Chemicals and Software

All of the chemicals and reagents used for polyphenols extraction and HPLC analyses were of analytical grade and obtained from reputable commercial suppliers. For example, from Merck (Mumbai, India), HPLC-grade methanol, ethyl acetate, petroleum ether, chloroform, acetonitrile, and acetic acid were obtained. Phenolic standards of analytical grades were purchased from Sigma Aldrich, St. Louis, MO, USA. Hi Media Laboratories, Mumbai, India, provided high-purity Folin–Ciocalteu’s phenol reagent, 1,1-diphenyl-2-picrylhydrazyl radical (DPPH), etc. The statistical software program Design Expert (version 13.0.5, Stat-Ease Inc., Minneapolis, MN, USA) was used to carry out the optimization study and perform the regression analysis of the experimental data and the descriptive statistics were analyzed using MS Excel (version 21).

## 4. Methods

### 4.1. Solvent Extraction

An amount of 100 mL solvent of each of the four different solvents (ethyl acetate, methanol, petroleum ether, and chloroform) was used to extract 10 g of powdered stem bark over the course of 48 hours at room temperature with continuous shaking. The extract was filtered and then it was dried in a vacuum using a rotary evaporator at a temperature of 45 °C. After drying, the extracts were collected in vials and preserved at a temperature of 4 °C until further analysis of total phenolic compounds and antioxidant activity.

### 4.2. Total Phenolic Content (TPC) Determination

Cicco et al.’s [56] method was applied to analyze the TPC of the *A. pavonina* extract with minor modifications. In a test tube, 100 µL of an aliquot of the plant extract in 1 mg/mL concentration was mixed with 100 µL of 1N Folin–Ciocalteu reagent and left for 2 min. Then, in the reaction, 800 μL of a 5% (*w/v*) sodium carbonate solution was added and the test tubes were incubated at 40 °C for 20 min in the absence of light. Against a blank, the absorbance of the test tube mixture was measured at 740 nm wavelength using a spectrophotometer (Shimadzu 04160). The amount of total phenolics was determined as mg of GAE/g using a standard curve ranging from 10 to 100 mg/mL concentration of gallic acid.

### 4.3. Determination of Antioxidant Activities (DPPH Radical Scavenging Method)

DPPH radical scavenging activity was assessed using a standard method as described by Thaipong et al. [57]. A total of 0.5 mL of plant extract of *A. pavonina* bark at different concentrations (20–100 μL per 1 mL methanol) mixed with 2 mL of DPPH methanol solution was kept in the dark for 24 h. Before measuring the absorbance at 517 nm, the mixture was rapidly stirred for 10 min. Ascorbic acid was used as a standard, and the results were expressed in IC_50_. The IC_50_ value of plant extract was the concentration at which it could scavenge 50% of the DPPH free radicals. IC_50_ value was calculated using a linear regression method. These measurements were performed in triplicate and the following equation was used to compute the proportion of radicals scavenged by DPPH (Equation (3)):

(3) $$\%\;\mathrm{DPPH}\;\mathrm{radical}\;\mathrm{scavenging}\;\mathrm{activity}=\left(A_{0}-\frac{A_{t}}{A_{0}}\right)\times 100$$

where A_0_ is the absorbance of the control (methanol instead of the plant extract), while At is the absorption value of the test substances (plant extract in different concentrations).

### 4.4. Selection of Relevant Variables and Experiment Design

In this study, the TPC extraction from the stem bark material of the *A. pavonina* tree and IC_50_ of the extract’s DPPH radical scavenging activity (antioxidant activities) were optimized using the response surface methodology (RSM). To improve the extraction efficiency, the central composite design (CCD) was adopted as an experimental design. The independent operational parameters, viz. solvent composition (*x*_1_, % *v/v* of methanol/water), extraction temperature (*x*_2_, °C), and extraction time (*x*_3_, h), were selected based on impact as indicated in earlier studies and were categorized into three levels: low (−1), medium (0), and high (+1) (Table 6). The experimental design comprised 20 combinations including six central points, generated using Design Expert software (version 13.0.5) (Table 1), and the experimental data were fitted to the following second-order polynomial equation (Equation (4)):

*y* = *b*_0_ + *b*_1_*x*_1_ + *b*_2_*x*_2_ + *b*_3_*x*_3_ + *b*_11_*x*_1_^2^ + *b*_22_*x*_2_^2^ + *b*_33_*x*_3_^2^ + *b*_12_*x*_1_*x*_2_+ *b*_13_*x*_1_*x*_3_ + *b*_23_*x*_2_*x*_3_ (4)

where y stands for the anticipated response; *b*_0_ indicates the value of the offset term; *b*_1_, *b*_2_, and *b*_3_ represent the linear coefficients; *b*_11_, *b*_22_, and *b*_33_ signify the quadratic coefficients; and *b*_12_, *b*_13_, and *b*_23_ imply the interaction coefficients. As per the extraction protocol suggested by the RSM model, 10 g bark powder was mixed with 100 mL of 71.61% (*v/v*) methanol/water and kept at 42.52 °C for 24 h under constant agitation in a shaker incubator. The extract was filtered before being vacuum-dried using a rotary evaporator. The dried extract was kept at 4 °C in an air-tight container until further analysis for identification and quantification of phenolic compounds employing the HPLC method.

### 4.5. Estimation of Phenolic Compounds via High-Performance Liquid Chromatography (HPLC)

An AGILANT 1260 (USA) device connected to the OpenLAB CDS 2.1 software data processing unit was employed for the evaluation of the phenolic compounds in the optimized extract of *A. pavonina* stem bark. A reversed-phase column, Luna C18 (25 cm length × 4.6 mm inner diameter × 5 µm thickness) (Phenomenex, Torrance, CA, USA), whose temperature was maintained at 28 °C, with a flow rate of the solvent set to 0.7 mL/min, was used for compound separation from a 20 µL plant sample and standard compound solution [19]. A standard compound solution with 1 mg/mL concentration was prepared by mixing 1 mg of standard compounds (kaempferol, gallic acid, syringic acid, chlorogenic acid, catechin hydrate, caffeic acid, p-coumaric acid, sinapic acid, coumarin, and quercetin) with 1 mL methanol solvent (HPLC-grade). The plant sample was prepared by mixing 1 mg of optimized extract of *A. pavonina* stem bark with 0.5 mL methanol solvent (HPLC-grade). Different proportions of solvent-B to solvent-A were used for gradient elution. For the first 28 min, the gradient of elution was altered using 10% to 40% of solvent-B in a linear manner, followed by 40% to 60% at 39 min, and 60 to 90% at 50 min. At 55 min, the mobile phase’s composition was restored to its original 10:90 (solvent-B/solvent-A) ratio, and the reaction was allowed to proceed for an additional 10 minutes before a new sample was injected. The total time required for analysis of each sample was 65 min. The chromatograms of the analyzed compounds were portrayed using a UV-Visible DAD detector, applying a range of wavelength from 280 to 320 nm. All the phenolic compounds were identified based on their retention time and by comparing them with standard compounds. For quantification of the identified compounds, a calibration curve was made against different concentrations of the respective standard compounds. 

## 5. Conclusions

In the present work, different solvents like ethyl acetate, methanol, petroleum ether, and chloroform were used to observe their role in the extraction of maximum TPC and to gain the utmost antioxidant activity. Among the tested solvents, methanol was identified as the most effective solvent for the extraction of phenolic substances. The applied RSM technique has successfully demonstrated the influence of various factors like solvent ratio, extraction temperature, and extraction time on extracting the highest TPC and gaining maximum antioxidant activity from *A. pavonina* stem bark. According to the RSM optimization analysis, the optimal conditions were a solvent composition of 71.61% (*v/v*) of methanol/water, an extraction temperature of 42.52 °C, and an extraction time of 24 h, respectively. Under these extraction conditions, the experimental values for the TPC and IC_50_ of the extract’s DPPH radical scavenging activity were found to be 181.69 ± 0.20 mg GAE/g dry tissue and 60.13 ± 0.11 mg/mL, respectively, which are very close to the theoretical projected values determined via RSM. Further, phenolic characterization using the HPLC technique revealed the presence of five phenolic acids and one coumarin compound with the highest quantities of chlorogenic acid (117.526 mg/g dry tissue) and sinapic acid (64.779 mg/g dry tissue), which demonstrated sensible antioxidant potential of the *A. pavonina* stem bark. This study highlighted the significance of applying RSM in maximizing the phenolic quantity and antioxidant activity obtained from the stem bark of *A. pavonina*, with a characterization of phenolics that validates that the stem bark of this plant is a rich source of phenolic compounds and a promising antioxidant agent for pharmaceutical industries and future drug development.

## Figures and Tables

**Table 1:** Experimental design and results obtained from the CCD design.

Run	Independent Parameters			Response Variables (*y*_1_ and *y*_2_)			
	*x*_1_ *	*x*_2_ **	*x*_3_ ***	Experimental Value		Predicted Value	
				TPC (mg GAE/g Dry Tissue)	IC_50_ of Extract’s DPPH Radical Scavenging Activity (mg/mL)	TPC (mg GAE/g Dry Tissue)	IC_50_ of Extract’s DPPH Radical Scavenging Activity (mg/mL)
1	40	20	1	115.54 ± 0.43	89.07±0.27	117.09	89.79
2	100	20	1	119.78 ± 0.42	88.25 ± 0.15	118.5	90.62
3	40	80	1	98.16 ± 0.71	92.11 ± 0.03	97.32	93.03
4	100	80	1	96.51 ± 0.036	90.14 ± 0.04	98.71	90.23
5	40	20	24	154.64 ± 0.39	79.91 ± 0.23	153.18	79.5
6	100	20	24	152.89 ± 0.11	78.33 ± 0.06	155.48	77.09
7	40	80	24	110.43 ± 0.30	89.89 ± 0.20	113.45	87.2
8	100	80	24	115.52 ± 0.41	82.2 ± 0.19	115.72	81.16
9	40	50	12.5	162.89 ± 0.15	78.2 ± 0.25	159.62	79.65
10	100	50	12.5	165.17 ± 0.16	77.23 ± 0.24	161.46	77.05
11	70	20	12.5	173.44 ± 0.13	76.85 ± 0.14	171.04	75.42
12	70	80	12.5	145.85 ± 0.10	76.35 ± 0.20	141.28	79.07
13	70	50	1	154.49 ± 0.07	69.43 ± 0.43	152.86	65.32
14	70	50	24	184.76 ± 0.02	50.26 ± 0.08	179.41	55.64
15	70	50	12.5	175.38 ± 0.16	67.44 ± 0.10	180.83	65
16	70	50	12.5	179.73 ± 0.22	66.62 ± 0.12	180.83	65
17	70	50	12.5	184.18 ± 0.20	61.83 ± 0.13	180.83	65
18	70	50	12.5	181.35 ± 0.18	62.52 ± 0.10	180.83	65
19	70	50	12.5	172.87 ± 0.43	70.06 ± 0.04	180.83	65
20	70	50	12.5	177.53 ± 0.41	64.04 ± 0.085	180.83	65

*x*_1_ *: solvent composition (% *v/v* of methanol/water); *x*_2_ **: extraction temperature (°C); *x*_3_ ***: extraction time (h); *y*_1:_ total phenolic content*; y*_2:_ IC_50_ of extract’s DPPH radical scavenging assay.

**Table 2:** ANOVA results for the response surface quadratic model developed for TPC.

Source	SS ^1^	df ^2^	MS ^3^	F-Value	*p*-Value	
Model	17,332.16	9	1925.80	85.75	<0.0001	significant
*x*_1_ *	8.48	1	8.48	0.3777	0.5526	
*x*_2_ **	2214.74	1	2214.74	98.62	<0.0001	significant
*x*_3_ ***	1762.52	1	1762.52	78.48	<0.0001	significant
*x*_1_²	1131.78	1	1131.78	50.39	<0.0001	significant
*x*_2_²	1673.92	1	1673.92	74.53	<0.0001	significant
*x*_3_²	593.59	1	593.59	26.43	0.0004	significant
*x*_1_ *x*_2_	0.0003	1	0.0003	0.0000	0.9971	
*x*_1_ *x*_3_	0.3828	1	0.3828	0.0170	0.8987	
*x*_2_ *x*_3_	199.30	1	199.30	8.87	0.0138	significant
Residual	224.58	10	22.46			
Lack of Fit	140.31	5	28.06	1.67	0.2947	not significant
Pure Error	84.27	5	16.85			
R^2^	0.98					
Adjusted R^2^	0.97					
Predicted R^2^	0.93					
Adequate precision	24.92					

^1^ SS: sum of square; ^2^ df: degree of freedom; ^3^ MS: mean square; * *x*_1_: solvent composition; ** *x*_2_: extraction temperature; *** *x*_3_: extraction time.

**Table 3:** ANOVA results for the response surface quadratic model developed for IC_50_ of extract’s DPPH radical scavenging activity.

Source	SS ^1^	df ^2^	MS ^3^	F-Value	*p*-Value	
Model	2337.24	9	259.69	20.66	<0.0001	significant
*x*_1_ *	16.98	1	16.98	1.35	0.2721	
*x*_2_ **	33.42	1	33.42	2.66	0.1340	
*x*_3_ ***	234.35	1	234.35	18.65	0.0015	significant
*x*_1_²	490.28	1	490.28	39.01	<0.0001	significant
*x*_2_²	412.49	1	412.49	32.82	0.0002	significant
*x*_3_²	56.13	1	56.13	4.47	0.0607	
*x*_1_ *x*_2_	6.59	1	6.59	0.5242	0.4856	
*x*_1_ *x*_3_	5.25	1	5.25	0.4176	0.5327	
*x*_2_ *x*_3_	9.95	1	9.95	0.7914	0.3946	
Residual	125.68	10	12.57			
Lack of Fit	75.50	5	15.10	1.50	0.3325	not significant
Pure Error	50.18	5	10.04			
R^2^	0.94					
Adjusted R^2^	0.90					
Predicted R^2^	0.72					
Adequate precision	14.92					

^1^ SS: sum of square; ^2^ df: degree of freedom; ^3^ MS: mean square; * *x*_1_: solvent composition; ** *x*_2_: extraction temperature; *** *x*_3_: extraction time.

**Table 4:** Predicted and experimental values under optimum conditions for model validation.

Parameters	Optimum Conditions	Predicted Values		Experimental Values		95% Confidence Interval			
						Low		High	
		TPC (mg GAE/g Dry Tissue)	IC_50_ of Extract’s DPPH Radical Scavenging Activity (mg/mL)	TPC (mg GAE/g Dry Tissue)	IC_50_ of Extract’s DPPH Radical Scavenging Activity (mg/mL)	TPC (mg GAE/g Dry Tissue)	IC_50_ of Extract’s DPPH Radical Scavenging Activity (mg/mL)	TPC (mg GAE/g Dry Tissue)	IC_50_ of Extract’s DPPH Radical Scavenging Activity (mg/mL)
*x*_1_ *	71.61	182.84	55.62	181.69 ± 0.20	60.13 ± 0.11	175.49	50.11	190.18	74.62
*x*_2_ **	42.52								
*x*_3_ ***	24.00								

* x_1_: solvent composition (%); ** *x*_2_: extraction temperature (°C); *** *x*_3_: extraction time (h).

**Table 5:** List of phenolic compounds identified in optimized stem bark extract of *A. pavonine* using HPLC analysis (*n* = 6).

Sl. No	Name of the Compound	Amount (mg/g Dry Tissue)	Chemical Group	Biological Activity
1	Gallic acid	0.042	Phenolic acid	Anti-inflammatory, antioxidant, anticancerous, antimicrobial [47,48]
2	Chlorogenic acid	117.526	Phenolic acid	Antioxidant, anti-inflammatory, anticancerous, antimicrobial [45]
3	Caffeic acid	5.809	Phenolic acid	Anti-inflammatory, antioxidant, antimicrobial, analgesic, and cardioprotective [49]
4	p-coumaric acid	0.487	Phenolic acid	Anti-inflammatory, antioxidant, antineoplastic, and antimicrobial [50]
5	Sinapic acid	64.779	Phenolic acid	Anti-inflammatory, antioxidant, anticancerous, and antimicrobial. [46]
6	Coumarin	1.523	Coumarins (benzopyrone)	Antimicrobial, antioxidant, anti-inflammatory, anticancer, anti-TB, and anticonvulsant [51,52]

**Table 6:** Actual and coded values of independent parameters were used in this study.

Symbols	Independent Variables	Unit	Coded Levels		
			−1 (Low)	0 (Medium)	+1 (High)
*x*_1_	Solvent composition (% *v/v* of methanol/water)	%	40	70	100
*x*_2_	Extraction temperature	°C	20	50	60
*x*_3_	Extraction time	h	1	12.5	24
**Dependent/Response Variables**				**Goal**	
*y*_1_	Total phenolic content	mg GAE/g dry tissue		Maximize	
*y*_2_	IC_50_ of extract’s DPPH radical scavenging activity	(mg/mL)		Maximize	

## Data Availability

All data are mentioned within the article.
